# Prevalence of smoking before and during pregnancy and changes in this habit during pregnancy in Northwest Russia: a Murmansk county birth registry study

**DOI:** 10.1186/s12978-016-0144-x

**Published:** 2016-03-08

**Authors:** Olga A. Kharkova, Alexandra Krettek, Andrej M. Grjibovski, Evert Nieboer, Jon Øyvind Odland

**Affiliations:** Department of Community Medicine, Faculty of Health Sciences, UiT The Arctic University of Norway, Tromsø, Norway; International School of Public Health, Northern State Medical University, office 1252, Troitsky avenue 51, Arkhangelsk, 163000 Russia; Department of Biomedicine and Public Health, School of Health and Education, University of Skövde, Skövde, Sweden; Department of Internal Medicine and Clinical Nutrition, Institute of Medicine, Sahlgrenska Academy at University of Gothenburg, Gothenburg, Sweden; Department of International Public Health, Norwegian Institute of Public Health, Oslo, Norway; Department of Preventive Medicine, International Kazakh-Turkish University, Turkestan, Kazakhstan; North-Easten Federal University, Yakutsk, Russia; Department of Biochemistry and Biomedical Sciences, Hamilton, ON Canada; School of Health Systems and Public Health, Faculty of Health Sciences, University of Pretoria, Pretoria, South Africa

**Keywords:** Smoking, Cigarettes, Smoking cessation, Pregnant women, Murmansk County Birth Registry, Russia, Arctic

## Abstract

**Background:**

Smoking during pregnancy leads to adverse maternal and birth outcomes. However, the prevalence of smoking among women in Russia has increased from < 5 % in the 1980s to > 20 % in the 2000s. We conducted a registry-based study in Murmansk County, Northwest Russia. Our aims were twofold: (i) assess the prevalence of smoking before and during pregnancy; and (ii) examine the socio-demographic factors associated with giving up smoking or reducing the number of cigarettes smoked once pregnancy was established.

**Methods:**

This study employs data from the population-based Murmansk County Birth Registry (MCBR) collected during 2006–2011. We used logistic regression to investigate associations between women’s socio-demographic characteristics and changes in smoking habit during pregnancy. To avoid departure from uniform risk within specific delivery departments, we employed clustered robust standard errors.

**Results:**

Of all births registered in the MCBR, 25.2 % of the mothers were smokers before pregnancy and 18.9 % continued smoking during pregnancy. Cessation of smoking during pregnancy was associated with education, marital status and parity but not with maternal age, place of residence, and ethnicity. Women aged ≤ 20–24 years had higher odds of reducing the absolute numbers of cigarettes smoked per day during pregnancy than those aged ≥ 30–34 years. Moreover, smoking nulliparae and pregnant women who had one child were more likely to reduce the absolute numbers of cigarettes smoked per day compared to women having ≥ 2 children.

**Conclusions:**

About 25.0 % of smoking women in the Murmansk County in Northwest Russia quit smoking after awareness of the pregnancy, and one-third of them reduced the number cigarettes smoked during pregnancy. Our study demonstrates that women who have a higher education, husband, and are primiparous are more likely to quit smoking during pregnancy. Maternal age and number of children are indicators that influence reduction in smoking during pregnancy. Our findings are useful in identifying target groups for smoking intervention campaigns.

## Background

Smoking during pregnancy is one of the most avoidable causes of adverse maternal and birth outcomes. Negative effects of maternal smoking include placental complications [1–3], reduced fetal growth [4–6], preterm birth [7], and low birth weight [8, 9]. Adverse effects of maternal smoking during pregnancy on the future health of a child can include child neurodevelopmental disorders, onset of childhood asthma, childhood overweight and obesity [10–12]. Intergenerational effects have also been reported [13]. Interestingly, maternal smoking during pregnancy has been associated with earlier onset of this practice in offspring [14].

Previous studies have identified maternal predictors for smoking during pregnancy [15–19]. Young mothers and less educated women exhibit an increased risk of doing so [15, 18] while marital partnership protects against adopting the smoking habit [16]. Furthermore, risk of continued smoking during pregnancy is enhanced among women who have had previous pregnancies than among nulliparous women [17, 20]. Whether alcohol intake during pregnancy is a predictor of discontinuing smoking is still debated. Dejin-Karlsson et al. [21] did not find an association while Giglia et al. [22] demonstrate that women who consume alcohol before pregnancy are more likely to stop smoking during pregnancy.

Many women stop smoking or reduce the amount of daily cigarettes when they become pregnant, or when planning a pregnancy [20, 23]. Hoekzema et al. [23] found that pregnant women have good knowledge about possible complications of smoking during pregnancy and a majority of smokers prefer to quit smoking gradually.

The prevalence of women smoking in Russia has varied from < 5 % in the mid-1980s to 12 % in the mid-1990s. During the beginning of 20^th^ century, the prevalence of smoking among Russian women ranged from 31.3 % in the 25–44 age group to 37.9 % in those aged 19–24 years [24]. To date, data for the prevalence of tobacco use among pregnant women in the Russian Federation is insufficient because of outdated results or cross-sectional studies with small sample size. The available prevalence rates of maternal smoking in Northwest and East Russia are: 16.3 % [25], 17.4 % [26] and 24.8 % [27]. Multinational cross-sectional web-based studies conducted in 15 countries suggest that 46.3 % Russian women are smokers and only 9.7 % of them continue to do so during pregnancy [28]. Others report smaller proportions, perhaps reflecting underreporting [29–31].

In the Russian Federation, maternal smoking during pregnancy has been shown to influence preterm birth [32], placental insufficiency [33], fetal growth [34], newborn adaptation [35] and anthropometric indices of newborns [36]. However, none of these studies examined how socio-demographic characteristics associate with smoking behavior during pregnancy. To improve the health of children, health workers should not only focus on the major determinants of maternal smoking, but also on cessation of this habit during pregnancy. In order to develop successful maternal smoking-cessation public health programs in Russia, knowledge about the socio-demographic characteristics of prospective mothers who quit or reduce smoking during pregnancy should be considered. To address this issue, we employed data from an established birth registry in Murmansk County, Northwest Russia to determine: (i) the prevalence of smoking before and during pregnancy; and (ii) the socio-demographic factors associated with discontinuing smoking or reducing the number of cigarettes smoked once pregnant.

## Methods

### Study setting and design

Our study focuses on Murmansk County, which is located in the northwestern part of the Russian Federation. It covers an area of 144,902 square km and borders on Norway and Finland. The population of Murmansk County was 766,281 on January 1^st^ 2015 [37].

We conducted a registry-based study with data from the MCBR. The MCBR is a joint effort of the University of Tromsø (Norway) and the Murmansk County Health Department (Russia). It was established in 2005, using the Norwegian Medical Birth Registry as a model [38]. Quality controls in 2006–2007 showed that the proportion of errors was < 1 % [38]. Our data include all pregnancies based on women attending antenatal clinics at the 15 delivery departments in Murmansk County during 2006–2011. The registry data were collected in hospitals and the number of births registered in the MCBR comprised 98.9 % of the official number of births recorded by the Health Department in Murmansk County. Details about its implementation and quality control have been described previously [38].

### Sample size

**Fig. 1:**
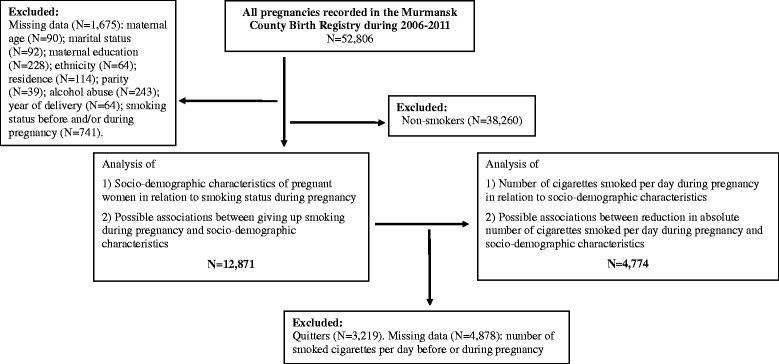
chart illustrating the selection of pregnant women

A total of 52,806 pregnancies were registered in the MCBR from January 1^st^ 2006 to December 31^st^ 2011. The sample size varies in some of our analyses, as missing or invalid data were excluded. Our study focused on 3 main components: (i) the socio-demographic characteristics and smoking status (*N* = 12,871); (ii) factors associated with smoking cessation during pregnancy (*N* = 12,871); and (iii) factors associated with smoking reduction during pregnancy (*N* = 4,774). The flow chart in Fig. 1 summarizes the participant exclusions.

### Data collection

Data on maternal age, residence, ethnicity, maternal education, marital status, parity, alcohol abuse, year of delivery, department of delivery, smoking before pregnancy, smoking during pregnancy and absolute number of cigarettes smoked per day both before and during pregnancy was based on medical records and the mothers themselves during interviews. Smoking-related information was self-reported.

### Dependent variables

The variable ‘smoking status during pregnancy’ was stratified into two subcategories: smoking before and during pregnancy (smokers), and smoking before, but not during pregnancy (quitters). Daily cigarette usage during pregnancy was converted into ordinal data (cigarettes/day) as light smokers (1–5), moderate smokers (6–10), and heavy smokers (≥11). Reduction in the number of cigarettes smoked per day after pregnancy awareness was dichotomized as “decreased” or “not decreased”. The latter included women who increased the number of cigarettes smoked per day during pregnancy, as well as those who did not change their smoking pattern.

### Independent variables

Maternal age was classified as: ≤ 19 years, 20–24 years, 25–29 years, 30–34 years and ≥ 35 years. Residence was defined as urban and rural. In terms of ethnic background, women were registered as either Russian or other. Education was categorized as incomplete secondary (0–9 years of schooling), secondary (10–11 years), vocational, university and unknown. Marital status was characterized as married, cohabitation or single (includes divorced or widowed). Parity was classified as 0, 1, and ≥ 2 deliveries. Alcohol abuse (based on documented evidence provided by physicians) was dichotomized into yes and no. Year of delivery was presented by the exact year. The data collection for the MCBR involved the delivery departments located at: Gadzievo, Sneznogorsk, Kola, Olenegorsk, Monchegorsk, Kovdor, Kirovsk, Aptity, Kandalaksha, Murmansk No.1, Murmansk No.2, Murmansk No.3, Nikel, Zaozersk and Severomorsk.

### Data analysis

We used Pearson’s chi-squared test to analyze categorical variables. Significance level for continuous non-normally distributed variables was based on Kruskal-Wallis and Mann–Whitney tests. By logistic regression we examined the relationships between socio-demographic characteristics of the women and smoking cessation during pregnancy, as well as the reduction in the absolute number of cigarettes smoked per day while pregnant. Crude and adjusted odds ratios (ORs) were calculated with 95 % confidence intervals (CI). To correct for any deviation from uniform risk within specific delivery departments, clustered robust standard errors were used. We tested for trends by entering ordinal variables as continuous term in regression analyses. The latter were carried out using SPSS version 22 (SPSS Inc., Chicago, IL) and STATA 13 statistical software.

### Ethical considerations

This study was granted ethical approval by the Ethical Committee of Northern State Medical University, Arkhangelsk, Russia, and the Norwegian Regional Committee for Medical and Health Research Ethics (REC-North), Tromsø, Norway.

## Results

### Smoking prevalence before and during pregnancy in Murmansk County

Of the 51,131 study participants, 25.2 % (95 % CI: 24.8–25.5 %) smoked before pregnancy, 18.9 % (95 % CI: 18.5–19.2 %) of these continued smoking during pregnancy. The overall proportion of women who smoked before pregnancy but stopped doing so once pregnant was 25.0 % (95 % CI: 24.3–25.8 %). Of those who smoked during pregnancy, 42.2 % were light smokers, 42.7 % were moderate smokers, and 15.1 % were heavy smokers. During pregnancy 1.0 % of smokers increased the number cigarettes per day, 62.1 % made no adjustment, and 36.8 % reduced their smoking frequency.

### Socio-demographic characteristics of women with different smoking status during pregnancy

The socio-demographic characteristics of the pregnant women are presented in Table 1. Smoking pregnant women were younger, had lower education, and were more likely to reside in rural areas. We found that smoking before and during pregnancy was more common in single women and those who were cohabiting. Furthermore, smoking before and during pregnancy was associated with alcohol abuse and multigravida (Table 1).

**Table 1:** Socio-demographic characteristics of pregnant women (*N* = 12,871) in Murmansk County, Northwest Russia, in relation to their smoking status during pregnancy

Variable	Number	Smoking before and during pregnancy *N* = 9,652 (%)	Smoked before, but not during pregnancy *N* = 3,219 (%)	*P*-value
Maternal age (years)				<0.001
≤ 19	1,513	12.8	8.8	
20–24	4,491	35.1	34.5	
25–29	3,788	28.6	31.9	
30–34	2,144	16.3	17.7	
≥ 35	935	7.3	7.1	
Residence				<0.001
Urban	10,565	80.6	86.6	
Rural	2,306	19.4	13.4	
Ethnicity				0.570
Russian	12,521	97.2	97.4	
Other	350	2.8	2.6	
Education				<0.001
Incomplete secondary	988	9.3	2.9	
Secondary	5,649	47.2	33.9	
Vocational	4,071	30.5	34.9	
University	2,099	12.3	28.2	
Unknown	64	0.7	0.1	
Marital status				<0.001
Married	7,503	53.7	72.1	
Cohabitation	3,239	27.8	17.2	
Single	2,129	18.5	10.7	
Parity				<0.001
0	7,380	55.6	62.4	
1	4,252	33.3	32.4	
≥ 2	1,239	11.1	5.2	
Alcohol abuse				<0.001
No	12,654	97.8	100.0	
Yes	217	2.2	0.0	

Calculated using the chi-squared test

A woman was more likely to continue smoking during pregnancy if she reported being a heavy smoker before pregnancy, compared to those who quit smoking after knowing they were pregnant (24.6 % vs 9.0 %; *p* < 0.001).

We found dissimilarity in the daily cigarette smoking frequency during pregnancy among women with different age, educational level, marital status, parity and status of alcohol abuse (Table 2). Pairwise comparison demonstrated that women aged ≥ 35 years and having incomplete secondary or secondary education smoked more cigarettes per day during pregnancy compared to women aged ≤ 19 years (*p* = 0.001) and those having university education (*p* < 0.001). Moreover, single pregnant women or women with a cohabitor, women with two or more previous deliveries and women abusing alcohol also smoked more daily during pregnancy than married women (*p* < 0.001), nulliparae or those having one child (*p* < 0.001) or without alcohol abuse (*p* < 0.001) (data not shown).

**Table 2:** Absolute numbers of cigarettes smoked per day during pregnancy in Murmansk County, Northwest Russia, in relation to socio-demographic characteristics of women (*N* = 4,774)

Variable	Number	Median	Q_1_–Q_3_^a^	*P*-value^b^
Maternal age (years)				0.017^c^
≤ 19	698	7	5–10	
20–24	1,767	7	5–10	
25–29	1,271	7	5–10	
30–34	743	7	5–10	
≥ 35	295	10	5–10	
Residence				0.248^d^
Urban	3,685	7	5–10	
Rural	1,089	7	5–10	
Ethnicity				0.433^d^
Russian	4,642	7	5–10	
Other	132	7	5–10	
Education				<0.001^c^
Incomplete secondary	497	8	5–10	
Secondary	2,545	8	5–10	
Vocational	1,369	7	5–10	
University	354	5	5–10	
Unknown	9	10	5–10	
Marital status				<0.001^c^
Married	2,562	6	5–10	
Cohabitation	1,406	10	5–10	
Single	806	10	5–10	
Parity				<0.001^c^
0	2,525	7	5–10	
1	1,667	7	5–10	
≥ 2	582	10	5–10	
Alcohol abuse				
No	4,671	7	5–10	<0.001^d^
Yes	103	10	10–20	

^a^ Q_1_–Q_3_ – first and third quartile

^b^ Calculated using ^c^ - Kruskal-Wallis test and ^d^ - Mann–Whitney test

### Factors associated with quitting smoking after pregnancy recognition

In the crude analysis, we found that quitting smoking during pregnancy was associated with maternal age, residence, education, marital status and parity but not ethnicity (Table 3).

**Table 3:** Associations between smoking cessation during pregnancy and socio-demographic characteristics of women (*N* = 12,871)

Variable	Crude OR^a^	95 % CI	*P*-value	Adjusted OR^a,b^	95 % CI	*P*-value
Maternal age (years)						0.083^c^
≤ 19	0.61	0.51–0.74	<0.001	0.97	0.75–1.24	
20–24	0.88	0.81–0.96	0.005	0.98	0.86–1.11	
25–29	1.00			1.00		
30–34	0.97	0.86–1.10	0.646	1.09	0.99–1.20	
≥ 35	0.88	0.72–1.06	0.172	1.16	0.94–1.43	
Residence						
Urban	1.00			1.00		
Rural	0.64	0.47–0.87	0.005	0.76	0.57–1.02	0.068
Ethnicity						
Russian	1.00			1.00		
Other	0.93	0.67–1.29	0.667	0.90	0.66–1.23	0.512
Education			<0.001^d^			<0.001^d^
Incomplete secondary	0.13	0.10–0.18		0.19	0.15–0.24	
Secondary	0.31	0.21–0.47		0.39	0.27–0.55	
Vocational	0.50	0.35–0.71		0.57	0.41–0.78	
University	1.00			1.00		
Unknown	0.09	0.04–0.19		0.12	0.06–0.25	
Marital status						
Married	1.00			1.00		
Cohabitation	0.46	0.33–0.64	<0.001	0.53	0.39–0.72	<0.001
Single	0.43	0.34–0.54	<0.001	0.49	0.41–0.58	<0.001
Parity			<0.001^c^			<0.001^c^
0	2.37	1.96–2.85		2.21	1.78–2.75	
1	2.05	1.71–2.45		1.69	1.46–1.95	
≥ 2	1.00			1.00		

^a^ Calculated using logistic regression with robust clustered standard errors by delivery department

^b^ OR adjusted for the variables listed in this table, alcohol abuse and delivery year

^c^ Test for linear trend

^d^ Test for linear trend (unknown category excluded)

After adjustment for confounders, the associations between maternal age, residence, ethnicity and quitting smoking were not significant. All other odds ratios listed in Table 3 were significant even after adjusting for confounders. Pregnant women with incomplete secondary, secondary, or vocational education had decreased odds of giving up smoking during pregnancy compared to women with university education.

We found that single pregnant women and those cohabiting were almost two-fold less likely to quit smoking during pregnancy than married women. Furthermore, nulliparae and pregnant women who had one previous delivery were more likely to stop smoking during pregnancy than multiparae. The adjusted odds of discontinuing smoking was 31.5 % less among rural pregnant women compared to the urban group (Table 3).

### Factors associated with reduction in the absolute number of cigarettes smoked per day during pregnancy

Crude analysis demonstrated a significant association between a reduction in number of cigarettes smoked during pregnancy and maternal age and parity. In both crude and adjusted logistic regression analyses, neither residence, ethnicity, education, nor marital status were significantly associated with the dependent variable (Table 4).

**Table 4:** Associations between reduction in the absolute number of cigarettes smoked per day during pregnancy and socio-demographic characteristics of women (*N* = 4,774)

Variable	Crude OR^a^	95 % CI	*P*-value	Adjusted OR^a,b^	95 % CI	*P*-value
Maternal age (years)						
≤ 19	1.16	0.93–1.45	0.180	1.14	1.01–1.28	0.035
20–24	1.18	1.02–1.36	0.027	1.14	1.02–1.26	0.018
25–29	1.00			1.00		
30–34	1.05	0.92–1.21	0.456	1.12	0.98–1.28	0.107
≥ 35	0.94	0.71–1.26	0.693	1.14	0.86–1.49	0.357
Residence						
Urban	1.00			1.00		
Rural	1.10	0.73–1.67	0.643	1.13	0.78–1.62	0.522
Ethnicity						
Russian	1.00			1.00		
Other	1.11	0.69–1.81	0.657	1.12	0.74–1.71	0.582
Education						
Incomplete secondary	0.68	0.40–1.15	0.151	0.72	0.47–1.11	0.139
Secondary	0.77	0.50–1.21	0.259	0.79	0.55–1.15	0.220
Vocational	0.77	0.58–1.02	0.074	0.78	0.61–1.02	0.068
University	1.00			1.00		
Unknown	0.38	0.11–1.31	0.126	0.40	0.11–1.50	0.176
Marital status						
Married	1.00			1.00		
Cohabitation	0.77	0.53–1.12	0.169	0.75	0.54–1.06	0.107
Single	0.78	0.55–1.12	0.181	0.75	0.55–1.03	0.078
Parity			<0.001^c^			<0.001^c^
0	1.63	1.33–2.00		1.62	1.36–1.93	
1	1.44	1.08–1.92		1.40	1.08–1.83	
≥ 2	1.00			1.00		

^a^ Calculated using logistic regression with robust clustered standard errors by delivery department

^b^ OR adjusted for the variables listed in this table, alcohol abuse and delivery year

^c^ Test for linear trend

After adjustment for covariates, younger pregnant women (aged ≤ 19–24 years) decreased the numbers of cigarettes smoked per day during pregnancy more frequently than women aged ≥ 25–29 years. Moreover, we found that smoking nulliparae and pregnant women who had one child were more likely to reduce the absolute numbers of cigarettes smoked per day compared to those having ≥ 2 children (Table 4).

## Discussion

Every fourth pregnant woman attending antenatal clinics during 2006–2011 at the 15 delivery departments in the Murmansk County reported smoking before pregnancy. Of these, one fourth stopped smoking during pregnancy. The overall rate of smoking before and during pregnancy in our study is close to Russian figures [24–27], but lower than in some European countries [28]. Pregnant women may stop smoking during pregnancy because of concerns about fetal and infant health [17]. We determined the proportion of quitters during pregnancy to be 25.0 %, which is less than in Australia [23], Spain [39] and the United States [40], but higher than in Denmark [41] and Greece [42]. Such differences may be related to variations in study design and sample selection, or the consequence of policy and social issues.

Our observations that smoking status before and during pregnancy was associated with a number of socio-demographic characteristics – namely maternal age, residence, education, marital status, and parity – are consistent with earlier studies [15–18, 20, 23].

The observation that the women in our study were more likely to continue smoking during pregnancy if they were heavy smokers suggests that the pre-pregnancy smoking level may serve as an indicator of addiction. Indeed, data from Australia demonstrate that smoking < 10 cigarettes per day in the pre-pregnancy period are more likely to interrupt this practice during pregnancy [22]. This might well be explained by motives to smoke. Russell [43] classifies smokers according to the predominant pattern of reinforcement. Those seeking sensory reward (e.g. taste, smell, observing the smoke), rather than pharmacological reward (i.e. stimulant effects of nicotine), are characterized by low nicotine intake and therefore are more likely to be able to stop smoking.

In our study older women, women with a low level of education, single women, those having ≥ 2 children and alcohol abusers smoked more during pregnancy than younger women, those having a high level of education, a husband or co-habiting partner, one previous child or none, and who did not abuse alcohol. Previous studies have also found that highly educated women exhibited increased odds of discontinuing smoking when pregnant [44, 45]. However, Smedberg et al. [28] suggest that the extent of smoking only differs significantly in relation to alcohol consumption during pregnancy.

We illustrate that selected socio-demographic characteristics constitute an indicator of smoking cessation during pregnancy in women in Murmansk County in contrast to other studies [28, 44]. We did not find an association between maternal age and odds of quitting smoking during pregnancy. As was suggested by Smedberg et al., the association between these variables after adjustment for potential confounders becomes non-significant [28]. However, Colman et al. illustrate that younger women are more likely to stop smoking during pregnancy compared to older women [44].

Our finding that women were more likely to quit smoking during pregnancy if they had no previous deliveries agrees with earlier findings [22, 42]. Moreover, we show a positive linear association between the number of previous deliveries and odds of quitting smoking during pregnancy. This may be explained by a women’s individual experience of giving birth to a healthy child despite smoking during pregnancy [28, 46].

Marital status has been extensively investigated as an indicator of smoking during pregnancy [18, 28, 47]. Our finding that single women and women with a cohabitor were twice less likely to quit smoking during pregnancy than those married has been interpreted to reflect a response to circumstances in women’s lives such as unsupportive partners [48].

Although rural women in our study smoked 1–5 cigarettes per day more often compared to urban women who smoked more heavily, rural women were less likely to quit smoking during pregnancy than their urban counterparts. A study from Greece suggests that the rural living is generally associated with lower smoking rates, which did not change during pregnancy [42].

A systematic review has demonstrated that to lessen the negative effects of smoking on pregnancy and fetal development, some women attempt to reduce their smoking rather than quit entirely [48]. In a literature review of 19 studies, 17 clearly demonstrate that more than half of all smoking women do not quit smoking completely during pregnancy [46]. These findings are consistent with our data that only one third of the pregnant women who smoked during pregnancy reduced the absolute numbers of cigarettes smoked. Moreover, older pregnant women and women with ≥ 2 children were less likely to reduce the number of cigarettes smoked than younger women and primipara, or those having one child.

Although common in other countries, studies like the current one are still lacking in Russia. Our examination of the socio-demographic determinants associated with reducing smoking or its cessation fills a void in Northwest Russia. We conclude that the socio-demographic characteristics identified to be related to alteration in smoking habits during pregnancy are similar between countries, despite cultural differences. Furthermore, we observed that for the marital status variable considered, which in the Russian tradition includes married, cohabitation and single, we found that only married women quit smoking during pregnancy.

An important strength of this study is that the data represent almost the total population of pregnant women attending antenatal clinics in Murmansk County during a defined time period. As indicated earlier, the data quality of the MCBR has been demonstrated to be excellent [38].

One limitation of our study is that the smoking information was based on self-reported data and we did not verify the use of tobacco by measuring biomarkers such as nicotine in the blood or saliva or cotinine in the urine [49]. This may have led to an underestimation of smoking rates, and thus would constitute measurement bias. However, we assessed smoking status before and during pregnancy during the first antenatal visit, which is likely to be more reliable than assessment after delivery [50]. Moreover, Giglia et al. [22] show that self-reported smoking status is a good measurement tool.

Another shortcoming pertains to missing data about the number of cigarettes smoked before and during pregnancy, as only half of the smokers provided this information. Furthermore, since our study only included women giving birth at the maternity clinics, the results may not be generalizable to women who gave birth outside such facility. Finally, the MCBR database did not allow us to explore potential confounders such as household income, maternal employment, paternal smoking status, maternal smoking during previous pregnancies, and relevant psychological factors as such data had not compiled. Interestingly, an earlier study has demonstrated that education is a more important factor in Russian perinatal epidemiology than employment and income [25].

## Conclusions

About 25.0 % of smoking women in Murmansk County in Northwest Russia stop smoking during pregnancy and one third reduced the amount of cigarettes smoked during pregnancy. Our study demonstrates that women, who have a higher education, husband, and are primiparous, are more likely to stop smoking during pregnancy. Maternal age and number of children are additional indicators that influenced the reduction in the absolute numbers of cigarettes smoked during pregnancy.

Our findings illustrate that selected socio-demographic characteristics of women who continue smoking during pregnancy will help in identifying target groups for future smoking intervention campaigns in Northwest Russia. Consistency of our findings with studies from other countries suggests that our analysis may also be applicable to the implementation of effective smoking cessation programs elsewhere in Russia.
